# Supplementary material for: The Obesity Amelioration Effect in High-Fat-Diet Fed Mice of a Homogeneous Polysaccharide from Codonopsis pilosula
Source: Molecules. 2022 Aug 22;27(16):5348. doi: 10.3390/molecules27165348 (PMC9415953; doi:10.3390/molecules27165348)
Supplement: Supplementary file 1 [file molecules-27-05348-s001.zip › molecules-1815898-supplementary.pdf]

molecules

Supplementary Materials

The Obesity Amelioration Effect in High-Fat-Diet Fed Mice of a Homogeneous Polysaccharide from *Codonopsis pilosula*

Qi Su [1,2,3,†], Jiangyan Huo [2,3,†], Yibin Wang [2,3,†], Yang Zhou [2,3], Dan Luo [2,3], Jinjun Hou [2,3], Zijia Zhang [2,3], Huali Long [2,3], Xianchun Zhong [2,3], Cen Xie [2,3], Min Lei [2,3,*], Yameng Liu [2,3,*] and Wanying Wu [1,2,3,*]

[1] School of Chinese Materia Medica, Nanjing University of Chinese Medicine, Nanjing 210023, China
[2] Shanghai Research Center for Modernization of Traditional Chinese Medicine, Shanghai Institute of Materia Medica, Chinese Academy of Sciences, Shanghai 201203, China
[3] National Engineering Laboratory for TCM Standardization Technology, Shanghai Institute of Materia Medica, Chinese Academy of Sciences, Beijing 100049, China
* Correspondence: mlei@simm.ac.cn (M.L.); yameng_liu@simm.ac.cn (Y.L.); wanyingwu@simm.ac.cn (W.W.)
† These authors contributed equally to this work.

Citation: Su, Q.; Huo, J.; Wang, Y.; Zhou, Y.; Luo, D.; Hou, J.; Zhang, Z.; Long, H.; Zhong, X.; Xie, C.; et al. The Obesity Amelioration Effect in High-Fat-Diet Fed Mice of a Homogeneous Polysaccharide from *Codonopsis pilosula*. *Molecules* **2022**, *27*, 5348. https://doi.org/10.3390/molecules27165348

Academic Editor: Fabio Sonvico

Received: 29 June 2022
Accepted: 10 August 2022
Published: 22 August 2022

Publisher's Note: MDPI stays neutral with regard to jurisdictional claims in published maps and institutional affiliations.

Copyright: © 2022 by the authors. Licensee MDPI, Basel, Switzerland. This article is an open access article distributed under the terms and conditions of the Creative Commons Attribution (CC BY) license (https://creativecommons.org/licenses/by/4.0/).

Abstract: A homogeneous polysaccharide coded as CPP-1 was extracted and purified from the root of *Codonopsis pilosula* (Franch.) Nannf. by water extraction, ethanol precipitation, and column chromatography. Its structure was analyzed by HPGPC-ELSD, HPLC, GC-MS, FT-IR, and NMR techniques. The results indicated that CPP-1 was composed of mannose (Man), glucose (Glc), galactose (Gal), and arabinose (Ara) at a molar ratio of 5.86:51.69:34.34:8.08. The methylation analysis revealed that the main glycosidic linkage types of CPP-1 were (1→4)-linked-Gal residue, (1→3)-linked-Glc residue, (1→3,4)-linked-Glc residue, (1→2,3,4)-linked-Glc residue, (1→)-linked-Glc residue, (1→)-linked-Man residue, and (1→)-linked-Ara residue. In vivo efficacy trial illustrated that CPP-1 supplements could alleviate HFD-induced mice obesity significantly, as well as improve obesity-induced disorders of glucose metabolism, alleviate insulin resistance, and improve the effects of lipid metabolism. The findings indicate that this polysaccharide has the potential for the treatment of obesity.

Keywords: *Codonopsis pilosula*; polysaccharide; structural characterization; obesity

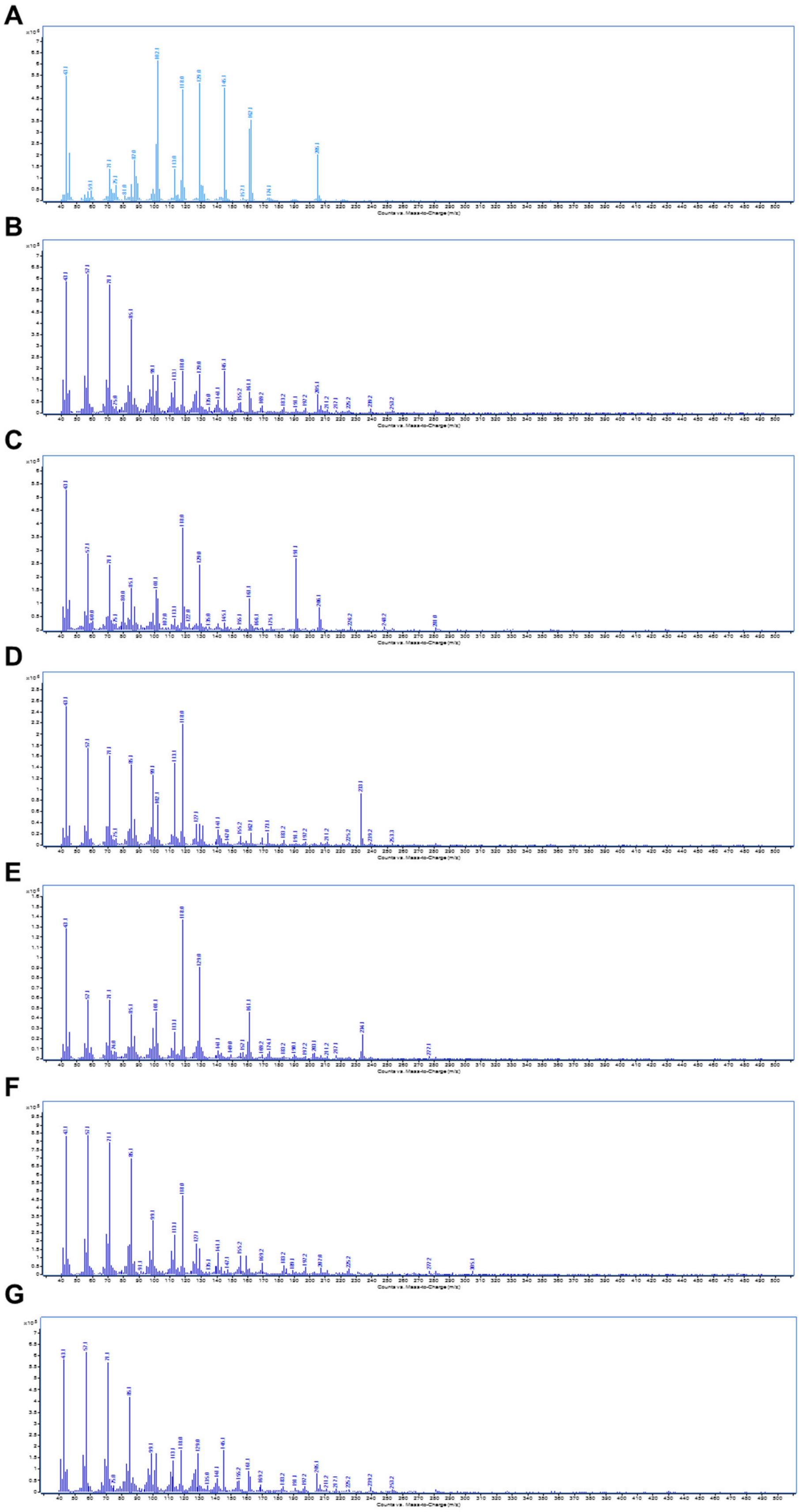

Figure S1. The ion fragments of different linkage patterns in GC/MS. (A) t-Glc(*p*), (B) t-Man(*p*), (C) t-Ara(*p*), (D) 1,4-Gal(*p*), (E) 1,3-Glc(*p*), (F) 1,3,4-Glc(*p*), (G) 1,2,3,4-Glc(*p*).
